# Supplementary material for: Development and Validation of a simple score for diagnosis of Leptospirosis at outpatient departments
Source: PLoS Negl Trop Dis. 2020 Jan 9;14(1):e0007977. doi: 10.1371/journal.pntd.0007977 (PMC6975559; doi:10.1371/journal.pntd.0007977)
Supplement: S1 Table — (DOCX) [file pntd.0007977.s001.docx]

**Supporting information**

**Table S1.** Univariable analysis of baseline demographic data, presenting symptoms, physical examinations and initial laboratory investigations of confirmed-cases and non-cases of Leptospirosis.

**Table S1.** Univariable analysis of baseline demographic data, presenting symptoms, physical examinations and initial laboratory investigations of confirmed-cases and non-cases of Leptospirosis.

| Clinical Characteristics | Confirmed cases  (n=82) | | Non-cases  (n=178) | | Crude OR  (95%CI) | P-value |
| --- | --- | --- | --- | --- | --- | --- |
|  | n | % | n | % |  |  |
| **Demographic** |  |  |  |  |  |  |
| Male | 59 | (72.0) | 129 | (72.5) | 0.97 (0.54-1.75) | 1.000 |
| Age, years (mean±SD) | 45.9 | ±14.6 | 47.6 | ±17.5 | 0.99 (0.98-1.01) | 0.432 |
| Life earning job |  |  |  |  |  |  |
| Agriculture | 54 | (65.9) | 118 | (66.3) | Ref | 0.693 |
| Employee | 9 | (11.0) | 11 | (6.2) | 1.79(0.70-4.57) | 0.225 |
| Government officer | 1 | (1.2) | 3 | (1.7) | 0.73(0.07-0.16) | 0.786 |
| Trader | 1 | (1.2) | 1 | (0.6) | 2.19(0.13-35.59) | 0.583 |
| Rubber cut | 1 | (1.2) | 2 | (1.2) | 1.09(0.10-12.31) | 0.943 |
| Fisherman | 0 | (0) | 2 | (1.1) | - | - |
| Others | 4 | (4.9) | 17 | (9.6) | 0.51(0.17-1.60) | 0.251 |
| > 1 jobs | 12 | (14.6) | 24 | (13.5) | 1.09(0.51-2.35) | 0.820 |
| Underlying conditions |  |  |  |  |  |  |
| None | 74 | (87.1) | 145 | (82.9) | 0.76(0.36-1.61) | 0.584 |
| HT | 5 | (5.9) | 13 | (7.4) | 0.82(0.28-0.39) | 0.799 |
| DM | 0 | (0) | 3 | (1.7) | - | 0.554 |
| Others | 9 | (10.6) | 22 | (12.6) | 0.87(0.38-1.99) | 0.839 |
| Onset duration, days (median, IQR) | 2 | 1, 3 | 3 | 1, 4 | 0.97(0.88-1.07) | 0.857 |
| **Symptoms** |  |  |  |  |  |  |
| Fever | 81 | (98.8) | 174 | (97.8) | 1.86(0.20-16.93) | 1.000 |
| Chill | 61 | (74.4) | 125 | (70.2) | 1.23(0.68-2.22) | 0.555 |
| Headache | 70 | (85.4) | 152 | (85.4) | 1.00(0.48-2.09) | 1.000 |
| Severe headache | 33 | (40.2) | 57 | (32.0) | 1.43(0.83-2.46) | 0.209 |
| Myalgia | 71 | (86.6) | 141 | (79.2) | 1.69 (0.82-3.52) | 0.172 |
| Calf pain | 58 | (70.7) | 112 | (62.9) | 1.42(0.81-2.50) | 0.262 |
| Jaundice | 7 | (8.5) | 6 | (3.4) | 2.68 (0.87-8.23) | 0.121 |
| Fatigue | 62 | (75.6) | 108 | (60.7) | 2.01 (1.12-3.61) | 0.024 |
| Nausea | 26 | (31.7) | 44 | (24.7) | 1.41(0.79-2.52) | 0.292 |
| Vomiting | 19 | (23.2) | 27 | (15.2) | 1.69 (0.87-3.25) | 0.120 |
| Diarrhea | 14 | (17.1) | 21 | (11.8) | 1.54(0.74-3.21) | 0.248 |
| Rhinorrhea | 7 | (8.5) | 23 | (13.0) | 0.62(0.26-1.52) | 0.404 |
| Cough | 41 | (50.0) | 100 | (56.18) | 0.78(0.46-1.32) | 0.422 |
| Hemoptysis | 2 | (2.4) | 2 | (1.13) | 2.19(0.31-15.81) | 0.593 |
| Breathing difficulty | 14 | (17.1) | 16 | (9.0) | 2.07 (0.96-4.48) | 0.093 |
| **Physical examinations** |  |  |  |  |  |  |
| Body temperature, °C (mean±SD) | 38.1 | ±1.1 | 37.9 | ±1.1 | 1.20 (0.94-1.54) | 0.141 |
| Pulse rate, per mins (mean±SD) | 100.4 | ±17.6 | 94.6 | ±16.2 | 1.02 (1.00-1.04) | 0.011 |
| SBP, mmHg (mean±SD) | 115.7 | ±20.2 | 120.7 | ±15.8 | 0.98 (0.97-0.99) | 0.033 |
| DBP, mmHg (mean±SD) | 69.5 | ±12.0 | 71.4 | ±10.5 | 0.98 (0.96-1.01) | 0.184 |
| Respiratory rate, per mins (mean±SD) | 21.4 | ±2.6 | 20.7 | ±2.0 | 1.15 (1.02-1.28) | 0.015 |
| Jaundice | 5 | (6.1) | 2 | (1.1) | 5.71 (1.08-30.10) | 0.035 |
| Conjunctival suffusion | 20 | (24.4) | 33 | (18.5) | 1.42(0.75-2.66) | 0.321 |
| Calf tenderness | 32 | (39.5) | 55 | (30.9) | 1.46(0.84-2.52) | 0.202 |
| Skin lesion | 17 | (20.7) | 44 | (24.7) | 0.80(0.42-1.50) | 0.531 |
| Hand | 7 | (8.5) | 19 | (10.7) | 0.78(0.31-1.94) | 0.663 |
| Foot | 14 | (17.1) | 25 | (14.0) | 1.26(0.62-2.57) | 0.576 |
| Leg | 10 | (12.2) | 17 | (9.6) | 1.32(0.57-3.01) | 0.518 |
| **Risk factors** |  |  |  |  |  |  |
| Flood or wet ground at home | 38 | (47.5) | 57 | (32.0) | 1.92 (1.23-3.30) | 0.025 |
| Flood or wet ground at workplace | 71 | (87.7) | 133 | (75.6) | 2.30 (1.09-4.84) | 0.031 |
| Contact animal water reservoir | 34 | (42.0) | 51 | (28.7) | 1.80 (1.04-3.12) | 0.045 |
| Animal contact | 64 | (79.1) | 131 | (74.0) | 1.32(0.70-2.49) | 0.437 |
| Skin wound/abrasion | 31 | (38.3) | 68 | (38.2) | 1.00(0.58-1.72) | 1.000 |
| **Laboratory findings** |  |  |  |  |  |  |
| Hb, g/dL (mean±SD) | 12.8 | ±1.7 | 12.9 | ±1.8 | 0.99(0.85-1.14) | 0.847 |
| Hct, % (mean±SD) | 39.9 | ±4.6 | 40.0 | ±5.0 | 0.99(0.94-1.05) | 0.809 |
| WBC, /µL (median, IQR) | 10,000 | 6,600, 12,200 | 7,900 | 5,700, 11,800 | 1.00 (0.99-1.00) | 0.049 |
| Neutrophil count ≥80% | 37 | (45.7) | 41 | (23.3) | 2.77 (1.58-4.85) | <0.001 |
| Neutrophil, %  (mean±SD) | 73.3 | ±14.1 | 68.3 | ±13.0 | 1.03(1.01-1.05) | 0.006 |
| Lymphocyte, %, (median, IQR) | 13.7 | 9.2, 23.6 | 19.9 | 13.1,27.3 | 0.97(0.94-0.99) | 0.003 |
| Monocyte, % (median, IQR) | 5.1 | 4, 8 | 7.1 | 5.6, 9.1 | 0.86(0.78-0.94) | <0.001 |
| Eosinophil, % (median, IQR) | 1.3 | 1, 3 | 2 | 1, 3.4 | 0.96(0.90-1.03) | 0.238 |
| Platelet, /µL (median, IQR) | 194,000 | 141,000, 271,000 | 214,500 | 181,500, 259,000 | 1.00(0.99-1.00) | 0.105 |
| eGFR, mL/min/1.73 m^2^ (mean±SD) | 85.8 | ±23.7 | 90.0 | ±22.9 | 0.99(0.98-1.00) | 0.187 |
| Urine Sp.gr. (median, IQR) | 1.02 | 1.02, 1.03 | 1.02 | 1.01, 1.03 | - | 0.068 |
| Urine pH % (median, IQR) | 5.5 | 5.5, 6.0 | 5.5 | 5, 6.5 | 0.85(0.59-1.21) | 0.866 |
| Urine glucose positive | 8 | (10.4) | 9 | (5.3) | 2.07(0.77-5.60) | 0.175 |
| trace | 3 | (3.9) | 1 | (0.6) | 7.00(0.72-68.48) | 0.094 |
| 1+ | 1 | (1.3) | 3 | (1.8) | 0.78(0.08-7.61) | 0.829 |
| 2+ | 1 | (1.3) | 0 | (0.0) | 1 | - |
| 3+ | 2 | (2.6) | 4 | (2.4) | 1.17(0.21-6.52) | 0.861 |
| 4+ | 1 | (1.3) | 1 | (0.6) | 2.33(0.14-37.84) | 0.551 |
| Urine protein positive | 44 | (57.1) | 65 | (32.2) | 2.15 (1.25-3.72) | 0.008 |
| trace | 14 | (18.2) | 31 | (18.2) | 1.44(0.68-3.02) | 0.339 |
| 1+ | 12 | (15.6) | 20 | (11.8) | 1.91(0.84-4.32) | 0.120 |
| 2+ | 12 | (15.6) | 8 | (4.7) | 4.8(1.80-12.67) | 0.002 |
| 3+ | 6 | (7.8) | 6 | (3.5) | 3.18(0.96-10.54) | 0.058 |
| Urine blood positive | 38 | (49.4) | 46 | (27.2) | 2.61 (1.49-4.56) | 0.001 |
| trace | 4 | (5.2) | 10 | (5.9) | 1.26(0.37-4.25) | 0.708 |
| 1+ | 14 | (18.2) | 19 | (11.2) | 2.32(1.07-5.06) | 0.034 |
| 2+ | 4 | (5.2) | 9 | (5.3) | 1.40(0.41-4.80) | 0.591 |
| 3+ | 16 | (20.8) | 8 | (4.7) | 6.31(2.51-15.86) | <0.001 |
| Urine bilirubin positive | 7 | (9.2) | 7 | (4.1) | 2.36 (0.80-6.99) | 0.137 |
| trace | - | - | - | - | - | - |
| 1+ | 3 | (4.0) | 7 | (4.1) | 1.01(0.25-4.03) | 0.986 |
| 2+ | 3 | (4.0) | 0 | (0.0) | - | - |
| 3+ | 1 | (1.3) | 0 | (0.0) | - | - |
| Urine ketone positive | 19 | (24.7) | 29 | (17.1) | 1.59 (0.83-3.06) | 0.169 |
| trace | 8 | (10.4) | 9 | (5.3) | 2.16(0.79-5.88) | 0.131 |
| 1+ | 4 | (5.2) | 10 | (5.9) | 0.97(0.29-3.23) | 0.964 |
| 2+ | 3 | (3.9) | 7 | (4.1) | 1.04(0.26-4.17) | 0.954 |
| 3+ | 4 | (5.2) | 3 | (1.8) | 3.24(0.70-4.94) | 0.131 |
| Urine leukocyte positive | 12 | (15.8) | 22 | (13.0) | 1.25 (0.58-2.68) | 0.555 |
| trace | 4 | (5.3) | 10 | (5.9) | 0.92(0.28-3.04) | 0.890 |
| 1+ | 4 | (5.3) | 5 | (3.0) | 1.84(0.48-7.07) | 0.376 |
| 2+ | 0 | (0.0) | 3 | (1.8) | - | - |
| 3+ | 4 | (5.3) | 4 | (2.4) | 2.30(0.56-9.47) | 0.250 |
| Urine urobilinogen positive | 18 | (23.7) | 38 | (22.4) | 1.08 (0.57-2.04) | 0.870 |
| trace | 12 | (15.8) | 30 | (17.7) | 0.91(0.44-1.90) | 0.803 |
| 1+ | 4 | (5.3) | 1 | (0.6) | 9.1(0.99-83.23) | 0.050 |
| 2+ | 0 | (0.0) | 4 | (2.4) | - | - |
| 3+ | 2 | (2.6) | 3 | (1.8) | 1.52(0.25-9.32) | 0.653 |
| Urine nitrite positive | 4 | (5.3) | 3 | (1.8) | 3.09 (0.67-14.17) | 0.207 |
|  |  |  |  |  |  |  |

Abbreviations: SBP, systolic blood pressure; DBP, diastolic blood pressure; WBC, white blood cell count; eGFR, estimated glomerular filtration rate; OR, odds ratio; SD, standard deviation; IQR, interquartile range.
