# Supplementary material for: Development and Validation of a simple score for diagnosis of Leptospirosis at outpatient departments
Source: PLoS Negl Trop Dis. 2020 Jan 9;14(1):e0007977. doi: 10.1371/journal.pntd.0007977 (PMC6975559; doi:10.1371/journal.pntd.0007977)
Supplement: Attachment — Submitted filename: Review PLoS NTD-PNTD-D-19-01397_TA.docx [file pntd.0007977.s002.docx]

**Review PLoS Neglected Tropical Diseases**

19^th^ September 2019

**Research manuscript:** “Development and validation of a simple score for diagnosis of leptospirosis at outpatient departments”

**Manuscript number:** PNTD-D-19-01397

Overall, the objective of this manuscript is relevant: there is a frequent overlap between bacterial and viral etiologies, and looking for simple point-of-care tools guiding health workers with the objective of identifying causes of fever among patients attending the first lines of care is critical.

Unfortunately, the study objective is undermined by several impediments which limits the clinical utility of this diagnostic score.

Here are my comments concerning this manuscript:

1. Overall, there are several typos and grammatically incorrect sentences alongside the manuscript. I suggest an English reviewer to read this manuscript prior any publication.
2. The review of a microbiologist would be welcome as well: for instance, the term “*Leptospira”* should use an italic font; in the Introduction section, evidence of *Leptospira* in the region should be further described based on values for prevalence/incidence among humans, as well as in the environment, with the corresponding diagnostic methods used (e.g. line 84, “…. highest in the northeastern region of the country”); the statement about MAT is incorrect, as one single acute sample over 1:800 (see 2013 CDC recommendations, (<https://wwwn.cdc.gov/nndss/conditions/leptospirosis/case-definition/2013/>) is sufficient to prove causality (line 95).
3. In the Method section, authors are using a MAT threshold of 1:400 in one single sample as a diagnostic confirmation, which is not what the latest guidelines recommend, especially in endemic areas where exposure is high.
4. The statistical analysis is unclear to me: authors calculated the sample size based on the difference of headache proportion between the two groups, if I understand well. This is very unusual. The calculation of an OR between these two groups with a corresponding coefficient would help to then measure an actual difference for all variables. Furthermore, the prevalence of headache directly impacted the measurement of the sample size, which prevents from fairly comparing the significance of other variables. All p-values and “adjusted” OR become more significant than the crude OR which makes me suspicious: For instance, all confirmed cases had significantly worse clinical characteristics than non-cases (e.g. systolic blood pressure; pulse rate; respiratory rate…), but it’s unclear if these variables have been included in the final model.
5. In the Methods section, it is unclear how clinicians initially suspected Leptospirosis, making the generalization of the results challenging. No clear inclusion criteria are provided/exist. In primary care, clinical judgment varies broadly, which questions how to apply this predictive score in other settings.
6. The study apparently aims to target patients with an undifferentiated fever. No clear definition exists to my knowledge. Also, the term “acute” should be defined. Do patients with a fever of less than 14 days were included, or only less than 7 days? Additionally, these “undifferentiated” patients did not seem to present with undifferentiated symptoms, as some of them presented with (non-undifferentiated) respiratory or digestive symptoms.
7. In the Results section, please provide p-values every time you are comparing proportions and percentages. The statement on lines 220-221 seems contradictive with previous significant p-values…Cases and non-cases do significantly vary for several clinical characteristics, which is unlikely to be linked to the detection of *Leptospira*. Non-cases seem less severe, especially regarding hemodynamic features (e.g. systolic blood pressure), and biological differences may therefore be due to a general difference in severity rather than to the presence of *Leptospira*.
8. Table 1 is presenting a very long series of symptoms that are non-significantly different between cases and non-cases. I would rather regroup symptoms by syndrome (e.g. respiratory for cough, dyspnea; digestive for nausea, vomiting, diarrhea) as authors were not powered to measure any difference for each symptom apart from headache. Moreover, I do not understand the absence of p-value for each occupation, drinking habits, or chief complaint.
9. Table 3: authors are using blood markers proportion instead of absolute values. This weakens the analysis to my view.
10. Authors are using mean (SD), and never use median (IQR): I’m wondering whether all continuous variables were normally distributed. This is very surprising to me.
11. Table 5 indicates performance increase in the diagnostic index when the score increases. I’m not sure I understand how the different scores are significantly different as their 95% CI overlap largely (0.64 [0.59], 0.67 [0.61-0.73]; 0.67 [0.61-0.74]). Overall, all AUCs (predictive values) remain low with an uncertain clinical utility. This should be acknowledged by the authors later in the Discussion.
12. The limitations should be elaborated: from patient recruitment, sample size calculation, model used, and diagnostic performance, many topics are uncovered.
13. Furthermore, some “non-cases” may be actual cases but remained undetected because of various reasons: for instance, non-cases seem less severe which lowers diagnostic techniques sensitivity; antibiotic prescription was higher in the non-cases even non-significantly, increasing the risk of false negatives; only half of patients had a convalescent sample for MAT, also increasing false negative cases…This undermines the robustness of the classification of the true negatives, and therefore the whole performance evaluation.
14. The conclusion is overstating the utility of the score (lines 390 and onwards). The mains limitations are not mentioned nor the need for studies to strengthen this first and initial evaluation. The term “validation” used by the authors for this score is an overstatement as well, as this study only provides a first and observational evidence. The design of the study indeed prevents from attributing any clear association between these indicators and the presence of *Leptospira*.
