# Supplementary material for: Development and Validation of a simple score for diagnosis of Leptospirosis at outpatient departments
Source: PLoS Negl Trop Dis. 2020 Jan 9;14(1):e0007977. doi: 10.1371/journal.pntd.0007977 (PMC6975559; doi:10.1371/journal.pntd.0007977)
Supplement: Attachment — Submitted filename: Response to reviewers_7Nov2019_NS.docx [file pntd.0007977.s003.docx]

Dear reviewers and editor of PLOS Neglected Tropical Diseases,

Subject: Submission of revised paper entitled **“Development and validation of a simple score for diagnosis of leptospirosis at outpatient departments”.**

The authors would like to thank the reviewers for commenting on our manuscript. Here are point-by-point responses, correction, along with additional analyses for the manuscript. Changes are highlighted in yellow color.

**Response to reviewers and editor**

**Reviewer#1:** No comment.

**Reviewer#2**

1.The authors should summarize Faine’s original and modified criteria for readers not familiar with these diagnostic scoring systems.

Response: A brief summary of both Faine’s original and modified criteria was inserted into the Introduction section, line 98-100

“Faine’s criteria or WHO score and modified version in 2004 and 2012, were developed as diagnostic guide for clinicians to make presumptive diagnosis of leptospirosis [21-24].”

and Discussion section, line 362-375.

“Faine’s criteria or WHO score was a diagnostic guide for clinicians to make presumptive diagnosis of leptospirosis since 1982. The criteria consisted of (1) clinical data such as headache, fever, muscle pain and jaundice (2) epidemiological factors such as contact with animals or known contaminated water and (3) laboratory findings such as isolation of Leptospira in culture or positive serology via microscopic agglutination test (MAT). The original score had been subsequently adapted to be applied in many health care settings worldwide. In 2004, a modified version was developed. The presence of rainfall and simple rapid tests e.g. ELISA and slide agglutination test (SAT) were included into the criteria…………”

2.The authors should summarize the sensitivity and specificity of Modified Faine’s Criteria as reported by others (references 24-27) and compare these published results with those reported here.

**Response:** We added a new summary Table (Table 6.) comparing the diagnostic indices of both Faine’s criteria and Modified Faine’s criteria with our newly derived score (Temeiam N et al.). The Tables also stated the detail of each study in terms of reference standard use, patient domain, study size including proportion of suspected and confirmed cases, and final recommendation of each paper. We hope this table would be informative enough to answer your comment.

3. The crude odds ratios for “wet ground at workplace” of 2.83 is not supported by the percentages in confirmed (27.4%) and non-cases (20.2%).

**Response:** For the univariable odds ratio of wet ground of workplace, we did recheck the calculation and the result already correct, [23x43 / 35x10 = 2.83], Table 2. However, in the revised manuscript, we decided to combine the wet ground and flood at workplace together for practicality during prediction. This did not affect the final model and the score development. The new crude odd ratio was highlighted in yellow in Table 2.

4.How much protein or blood in the urine is required for a positive score for those criteria?

**Response:** For both urine protein and urine blood dipstick test result, the reported results of trace or more (1+, 2+, 3+, and 4+) were considered positive. As for early disease, any trace amount of protein and haemoglobin should be considered significant. This statement was added at Material section, line 191-192.

5.The data for patients with neutrophil percentage >80% among confirmed and non-cases is missing.

**Response:** We added the data showing the proportions of patients with neutrophil count more than 80% in Table 1 and highlighted in yellow. The comparison of these proportions was done with exact probability test and the odds ratio was derived via univariable logistic regression.

6.The data for some of the modified Faine’s criteria (such as rainfall) among confirmed and non-cases is missing.

**Response:** As our study did not primarily intended to calculate the modified Faine’s criteria (2004), the variable ‘rainfall’ was not collected. However, we can calculate the original Faine’s criteria or WHO score from our dataset which we believed was more scientific and reliable than Faine’s criteria (Table 5). In addition, the variable “rainfall” might not add any predictive ability to the model as history of rainfall without stagnation of water would not increase risk of a person to exposure of pathogenic *Leptospira*.

7. What percentage of non-cases were diagnosed with infections other than leptospirosis?  What were those alternative diagnoses and how often did they occur?

**Response:** The percentage of non-cases was 68.5%. The confirmation of diagnosis, other than Leptospirosis, in non-cases was not performed. We added this limitation statement at Method section, line 200-201, and Discussion section, the limitation paragraph, line 460-464.

8. Did any of the leptospirosis patients in this study develop more severe complications including death?  How many of the leptospirosis cases in this study were later admitted to the hospital or treated for leptospirosis?

**Response:** Of 82 leptospirosis confirmed cases, 43 cases (52.4%) were treated as outpatient treatment and appointed for subsequent follow-up visit. Thirty cases (40.2%) were admitted for in-patient hospital care. Six cases (7.3%) were defined as severe leptospirosis and were transferred to higher level hospital. However, none of the patient had lethal complication. We added these statements in Result section, line 281-284.

9.What was the average time from onset of symptoms to evaluation?

**Response:** The average onset of fever in this cohort of patients was described as follow:

i. Mean onset of fever was 3.15 days with standard deviation of 3 days
ii. Median onset of fever was 2.5 days with IQR of 1 and 4 days, respectively.
iii. The mean onset of fever in confirmed cases was 3.0±2.3
iv. The mean onset of fever in non-cases was 3.2±3.3
v. Statistical comparison did not show statistically significant result.

We added this data at Result section, line 289-290.

**Reviewer#3**

1.Most methods were lacking of reference citations for example, all laboratory tests used in the study such as culture, polymerase chain reaction, and MAT methods; cases and non-cases definitions. Section "Blood sample and specimen collection" is not well described. For example, which kinds of blood tubes were used for culture, PCR and MAT, which parts of blood (or urine) samples were collected after centrifuge and for what tests, and centrifugation force was not specified. It is confusing that frozen samples were sent to culture (this definitely need the step-by-step-protocol or reference citation.

**Response:** Thank you very much for this important point. We have added this part in Method section, line 168-188 as the following sentences

“On the first day of enrollment, 12 ml of blood and 30 ml of urine sample were collected from the patients. Blood samples for laboratory investigations other than culture were collected in EDTA tubes and clotted blood tubes. Both plasma and urine sample were centrifuged at 3000g for 10 minutes and were subsequently frozen at -20 degree Celsius until being transferred to the central laboratory. Both samples were stored at -80 degree Celsius until they were taken for analysis. For direct culture of *Leptospira*, a drop of blood and urine was separately inoculated into 4 mL of liquid Ellinghausen-McCullough-Johnson-Harris (EMJH) at 29 degree Celsius for 14 days. Detection for *Leptospira* was done with direct observation via dark field microscopy. For real time PCR, 200 µL of whole blood was sampled from EDTA tube. A high Pure PCR template preparation kit (Roche Diagnostics, Germany) was used for DNA extraction with 50 µL elution buffer. The amplification primers for LipL32 gene were LipL32-45F (5’-AAG CAT TAC CGC TTG TGG TG-3’) and LipL32-286R (5’-GAA CTC CCA TTT CAG CGA TT-3’). The fluorescent probe was LipL32-189P (FAM-5’-AA AGC CAG GAC AAG CGC CG-3’-TAMRA). The PCR reactions of the samples were performed in a final volume of 20 µL which contained 5 µL of genomic DNA and 15 µL of reaction mix (10 µL of 2X TaqMan Universal PCR Master Mix (Applied Biosystems, Foster City, CA), 1 microliter of each 10 µM primer, 0.4 µL of 10 µM probe under 2.6 µL distilled water). The real time PCR program consisted of 45 cycles, each consisting of 95 degree Celsius for 15 seconds and 60 degree Celsius for one minute. Positive and negative controls were included in every experiment done. Results were read by threshold cycle (Ct) value. Microscopic agglutination test or MAT was performed as described in the standard protocol of the World Health Organization (WHO) guideline. A positive MAT was defined as a single serum cut-point of ≥1:800 based on confirmed laboratory diagnosis by CDC definition 2013.

2.What is the method used for sample size estimation?

**Response:** For development of clinical prediction rules, there is currently no standard approach for estimation of study size. We reviewed the unpublished data and patient records comparing the clinical characteristics of leptospirosis confirmed cases and non-cases at Si Sa Ket hospital during 2015. The proportion of patients reported exposure to contaminated water was 0.73 and 0.25 for confirmed cases and non-cases of leptospirosis, respectively. Using the comparison of two proportions approach, 12 confirmed cases and 47 non-cases were needed to achieve 80% statistical power and a two-sided alpha error of 0.05. A 10-events-per-variable rule of thumb was suggested by many literatures including the TRIPOD statements for reporting of clinical prediction rules development. For our study, as we planned to include at least 5 potential predictors within the final model, at least 50 confirmed cases were required for model derivation. At confirmed cases: non-cases ratio of 1:4, this study planned to recruit at least 250 patients (50:200). We added this part at the Method section, line 216-227.

3. Was the modified WHO score used in the study?

**Response:** Our study was not based on both the original Faine’s criteria or WHO score and the modified version of WHO score. Our dataset contained enough variable for creating the new score which we called “OPD Lepto Score”.

4. For the definition of leptospirosis cases, the first criteria "isolation of Leptospira from clinical specimen" might not accurate. How much do authors be certain if pathogenic Leptospira species isolated from urine samples collected from patients who having 2 days of fever? Or how much author be certain the species or group (pathogens or non-pathogens) of Leptospira isolated from blood or urine specimens.

**Response:** For all isolation of leptospira, both from blood and urine (in our study, only blood culture positive, not the urine), we have confirmed all the isolation by performed the 16S rRNA sequencing. All the sequencing revealed leptospira interrogans. We have added this statement in Material and Method section, line 176-177, and line 196-197.

5. Clinical outcomes (at least after 7 days, the second visit) and treatments are important for evaluation the success of non-intervention routine practices. However, these data were not collected in this study.

**Response:** We did collect the clinical outcome until the second visit. We have summarized the clinical outcome in Result section, line 281-284.

“Forty-three cases (52.4%) were treated as outpatient and appointed for subsequent follow-up visit. Thirty cases (40.2%) were admitted for in-patient hospital care. Six cases (7.3%) were defined as severe leptospirosis and were transferred to higher level hospital. However, none of the patient had lethal complication.

6. The results of Leptospira isolations (both blood and urine specimens), PCR and MAT (paired serum) were not presented. These results are keys results for proper "cases" and "non-cases" definitions. Only final number of cases and non-cases were described.

**Response:** We agree with the reviewer and provide the detail of each confirmatory test. From 82 cases of leptospirosis confirmed cases, 63 cases (76.8%) had blood PCR positive, 37 cases (45.1%) had MAT titer higher than 1:800, 11 cases (14.9%) had blood culture positive, and no case for urine culture positive. We have re-designed the patient flow diagram in Figure 1.

7.The results of WHO score in each "cases" and "non-cases" groups are important, however, they were not shown.

**Response:** We added a new table (Table 5) comparing between our newly derived OPD score and the original Faine’s criteria or WHO score in terms of score distribution across confirmed cases and non-cases, with mean, standard deviation and predictive ability via positive likelihood ratio.

**Table 5.** Score categorization and likelihood ratio of positive (LHR+) in OPD Lepto score and Faine’s score

| Probability  Categories | Score | Confirmed cases  (n=75) | | Non-cases  (n=167) | | LHR+ | (95%CI) | P-value |
| --- | --- | --- | --- | --- | --- | --- | --- | --- |
|  |  | N | % | n | % |  |  |  |
| OPD Lepto Score |  |  |  |  |  |  |  |  |
| Low | <3.5 | 21 | 28.0 | 103 | 61.7 | 0.45 | (0.31-0.67) | <0.001 |
| High | ≥3.5 | 54 | 72.0 | 64 | 38.3 | 1.88 | (1.48-2.39) | <0.001 |
| Mean±SD | - | 4.2 | ±1.5 | 3.0 | ±1.6 | - |  | <0.001 |
| Conventional Faine’s score |  |  |  |  |  |  |  |  |
| Low | <26* | 97.3 | 76.0 | 165 | 98.8 | 0.99 | (0.95-1.03) | 0.407 |
| High | ≥26* | 2 | 2.7 | 2 | 1.2 | 2.23 | (0.32-15.5) | 0.407 |
| Mean±SD | - | 19.55 | ±3.2 | 17.7 | ±4.01 | - |  | 0.002 |

8.Table 1 and 3 shows combination of both category parameters and numeric parameters. However, the heading are not specify well.

**Response:** We have rewritten the heading of Table 1 and Table 3. As our original manuscript contained lots of tables and might be considered redundant, we re-designed the presentation of our tables by presenting only the relevant baseline clinical characteristics, physical examinations, exposure of risk factors, and laboratory investigations that would be included in the multivariable logistic model for model derivation in only one table (Table 1). Other factors without statistical significance from univariable analysis (P-value>0.2) were not shown in the revised version of the manuscript. However, the detailed tables of all characteristics would be reported in the supplementary appendix (newly added: Supplement Table 1).

9.Is it accurate to use mean (instead of median and IQR) to compare the OPD score of "cases" and "non-cases" group as shown in Table 6?

**Response:** The distribution of OPD score and conventional Faine’s score for both confirmed-cases and non-cases groups was normally distributed according to visualization via histogram. The standard deviation of both groups was also similar and much smaller than the means.

The assumption of comparison of two independent means via independent t-test would hold in this scenario (independency, normal distribution and equality of variances). For this reason, we believed that the presentation of mean, in consistent with the statistical test that we used was reasonable and did not violate any statistical assumption.

10. Is the OPD score suitable for human leptospirosis with other complication such as respiratory or heart diseases (not only kidney complication). Or is the OPD score is suitable for other endemic area in the Southern of Thailand or Malaysia, where less cattle farms and rice paddy fields but more rubber gardens, forest trekking and stray dogs.

**Response:** The study domain of the OPD score, or “intended to be diagnosed” population was for patients with early onset and mild or vague symptoms. Thus, for the patients with frank symptoms or the patients with complications which required hospital admission, the OPD score might not be at good use and should not be applied. We emphasized this limitation in Discussion section, line 442-446.

Regarding for the question concerning the suitability of the OPD score for other endemic area such as the southern part of Thailand and Malaysia, we could not confidently answer this until the future external validation study be conducted within those regions. As the different in environments, occupation of local people could be an important effect modifiers of the score performance.

11.Revision the "Methods" with all possible reference citations and laboratory brief protocols. And also specify the following details; PCR format (conventional or real-time PCR), target gene (rrs, lipL32 or others), Laboratory institute where all tests performed. Adding PCR, culture and MAT results; and the WHO score results. Improving heading and legend in Table 1 and 3. Using median and IQR instead of mean and SD when it is appropriate.

**Response:** We have revised the Method section as reviewer ‘s suggestion, detail of real time PCR format, line 176-188. We also added the PCR, culture, MAT results in Figure 1, and the WHO score results in Table 5. We improved heading and legend in Table 1 and Table 3. We also rechecked the statistical analysis as mentioned in response to Question 9.

11.Improving discussion on the limitation of the "OPD score".

**Response:** We have revised the limitation of “OPD score” in Discussion seciton as suggestion of reviewer.

12.Checking spelling and grammar for the whole manuscript again. Please correct the "drip stick". It should be "dipstick".

**Response:** The spelling and grammar was rechecked and corrected as suggested.

**Editor’s comment**

1.The review of a microbiologist would be welcome as well: for instance, the term “*Leptospira”* should use an italic font; in the Introduction section, evidence of *Leptospira* in the region should be further described based on values for prevalence/incidence among humans, as well as in the environment, with the corresponding diagnostic methods used (e.g. line 84, “…. highest in the northeastern region of the country”); the statement about MAT is incorrect, as one single acute sample over 1:800 (see 2013 CDC recommendations, (<https://wwwn.cdc.gov/nndss/conditions/leptospirosis/case-definition/2013/>) is sufficient to prove causality (line 95).

**Response:** All “*Leptospira*” within the manuscript were changed to italic font. The description of incidence and prevalence of leptospirosis within the region was revised for comprehension, line 79-84. The statement concerning MAT was updated according to the recent definition of CDC 2013 as suggested line 191-192.

2.In the Method section, authors are using a MAT threshold of 1:400 in one single sample as a diagnostic confirmation, which is not what the latest guidelines recommend, especially in endemic areas where exposure is high.

**Response:** The statement concerning MAT was updated according to the recent definition of CDC 2013 as suggested, line 1880-191.

3.The statistical analysis is unclear to me: authors calculated the sample size based on the difference of headache proportion between the two groups, if I understand well. This is very unusual. The calculation of an OR between these two groups with a corresponding coefficient would help to then measure an actual difference for all variables. Furthermore, the prevalence of headache directly impacted the measurement of the sample size, which prevents from fairly comparing the significance of other variables. All p-values and “adjusted” OR become more significant than the crude OR which makes me suspicious: For instance, all confirmed cases had significantly worse clinical characteristics than non-cases (e.g. systolic blood pressure; pulse rate; respiratory rate…), but it’s unclear if these variables have been included in the final model.

**Response:** For development of clinical prediction rules, there is currently no standard approach for estimation of study size. The authors reviewed the unpublished data and patient records comparing the clinical characteristics of leptospirosis confirmed cases and non-cases at Si Sa Ket hospital during 2015. The proportion of patients reported exposure to contaminated water was 0.73 and 0.25 for confirmed cases and non-cases of leptospirosis, respectively. Using the comparison of two proportions approach, 12 confirmed cases and 47 non-cases were needed to achieve 80% statistical power and a two-sided alpha error of 0.05. A 10-events-per-variable rule of thumb was suggested by many literatures including the TRIPOD statements for reporting of clinical prediction rules development. For our study, as we planned to include at least 5 potential predictors within the final model, at least 50 confirmed cases were required for model derivation. At confirmed cases: non-cases ratio of 1:4, this study planned to recruit at least 250 patients (50:200). We added these statement in Method section, line 217-228.

When calculating the study size using the comparison of proportions approach, only two proportions were needed. Anyway, the odds ratio could be calculated but the concept behind the estimation remains the same. We believed that any approach would yield insignificant differences. For derivation of predictive model via multivariable logistic regression, the number of events is the key for stable model and prevention of model overfitting.

As multivariable analysis was used for model derivation, the adjusted odds ratio could become more or less significant depending on the underlying dataset. For clarity, we decided to revise the presentation table for our modeling procedure. In Table 2, the multivariable odds ratios of both full and reduced model (via stepwise backward elimination) were shown.

Potential predictors were pre-selected from literature reviews and previously reported scoring system and diagnosis criteria. All predictors were explored for significant difference using univariable analysis with statistical criteria of P-value<0.2. Any predictor with p-value less than the critical value would be chosen for inclusion in the multivariable analysis. Finally, a total of 27 predictors that surpass the statistical criteria were included and subsequently eliminated.

4.In the Methods section, it is unclear how clinicians initially suspected Leptospirosis, making the generalization of the results challenging. No clear inclusion criteria are provided/exist. In primary care, clinical judgment varies broadly, which questions how to apply this predictive score in other settings.

**Response:** Clinicians suspected the diagnosis of Leptospirosis based on classical clinical syndrome stated in the classic WHO clinical criteria such as the presence of acute febrile illness (onset of fever less than 14 days), headache, myalgia and history of exposure to animal water reservoirs or flooded environments either at home or at work. It is, however, important to note that the degree of suspicious might vary across different season and endemic areas.

We inserted the “pragmatic clinical criteria” within the study participants part, Method section, for clarity, line 140-144.

5.The study apparently aims to target patients with an undifferentiated fever. No clear definition exists to my knowledge. Also, the term “acute” should be defined. Do patients with a fever of less than 14 days were included, or only less than 7 days? Additionally, these “undifferentiated” patients did not seem to present with undifferentiated symptoms, as some of them presented with (non-undifferentiated) respiratory or digestive symptoms.

**Response:** This study included the patients who presented with acute febrile illness with onset of fever less than 14 days and were suspected of having Leptospirosis based on clinical syndrome such as headache, myalgia, and conjunctival suffusion with history of exposure to possible risk factors, line 140-144. Mean onset of fever was 3.15 days with standard deviation of 3 days. Median onset of fever was 2.5 days with IQR of 1 and 4 days, respectively. There were 5 patients with onset of fever day more than or equal to 14 days; However, only 1 of them was confirmed of having leptospirosis (onset of fever=14 days).

6.In the Results section, please provide p-values every time you are comparing proportions and percentages. The statement on lines 220-221 seems contradictive with previous significant p-values…Cases and non-cases do significantly vary for several clinical characteristics, which is unlikely to be linked to the detection of *Leptospira*. Non-cases seem less severe, especially regarding hemodynamic features (e.g. systolic blood pressure), and biological differences may therefore be due to a general difference in severity rather than to the presence of *Leptospira*.

**Response:** Thank you very much for these comments. We inserted p-values for every comparison reported in the result sections as suggested. We cutted off the confusing statement on lines 220-221 and revised the reporting of results in the revised version of the manuscript, line 292-298. From our data, it was evident that patient with leptospirosis had some severe clinical characteristics such as fatigue, low systolic blood pressure, high pulse rate, high respiratory rate, and jaundice, even in the early phase of the disease compared to other local tropical diseases (non-cases).

7.Table 1 is presenting a very long series of symptoms that are non-significantly different between cases and non-cases. I would rather regroup symptoms by syndrome (e.g. respiratory for cough, dyspnea; digestive for nausea, vomiting, diarrhea) as authors were not powered to measure any difference for each symptom apart from headache. Moreover, I do not understand the absence of p-value for each occupation, drinking habits, or chief complaint.

**Response:** As our original manuscript contained lots of tables and might be considered redundant, we re-designed the presentation of our tables by presenting only the relevant baseline clinical characteristics, physical examinations, exposure of risk factors, and laboratory investigations that would be included in the multivariable logistic model for model derivation in only one table (Table 1). Other factors without statistical significance from univariable analysis (P-value>0.2) were not shown in the revised version of the manuscript. However, the detailed tables of all characteristics would be reported in the supplementary appendix, Table S1 (newly added).

8.Table 3: authors are using blood markers proportion instead of absolute values. This weakens the analysis to my view.

**Response:** We thank the editor for this valuable point. We treated the percentage of neutrophils, lymphocytes, monocytes and eosinophils as continuous values and presented them as mean and standard deviation or median and interquartile range as appropriate. In practice, the result of complete blood count or CBC were reported in the form of percentage of each blood components and clinicians usually interpreted these values directly.

9.Authors are using mean (SD), and never use median (IQR): I’m wondering whether all continuous variables were normally distributed. This is very surprising to me.

**Response:** We rechecked the distribution of all continuous variables and chose the appropriate summarize statistics as suggested. The statistical test for comparison was also chosen based on the distribution of the underlying data, t-test for normally distributed data and ranksum (Man-Whitney U test) test for non-normally distributed data.

10. Table 5 indicates performance increase in the diagnostic index when the score increases. I’m not sure I understand how the different scores are significantly different as their 95% CI overlap largely (0.64 [0.59], 0.67 [0.61-0.73]; 0.67 [0.61-0.74]). Overall, all AUCs (predictive values) remain low with an uncertain clinical utility. This should be acknowledged by the authors later in the Discussion.

**Response:** We thank the Editor for this suggestion. Our newly derived score was developed primarily for outpatient clinical setting, where patients usually presented with mild and vague clinical syndrome. Thus, comparing to previously derived score such as Faine’s criteria and modified Faine’s criteria which were all derived from hospitalized cohort of patients, our score theoretically and practically (as shown from the comparison of AUC with the classical Faine’s criteria (0.72 vs 0.62, p=0.014)) could overcome other score in terms of distinguishing early leptospirosis patients who visited “outpatient” clinic during their early phase of the disease. Nonetheless, the fair discriminative performance via AUC criteria will be acknowledged in the Discussion section as suggested, line 452-456.

11.The limitations should be elaborated: from patient recruitment, sample size calculation, model used, and diagnostic performance, many topics are uncovered.

**Response:** We rewrote the limitations part, Discussion section as suggested.

“However, there were some limitations need to be addressed. First, the study protocol planned to schedule all enrolled patients for 2^nd^ visit for blood samples for MAT to observe and interpret four-fold rising of convalescent titers, but only 48.9% of patients came for the blood tests. Thus, the number of confirmed cases could be underestimated in some degree. Second, distinguishing a group of patients at their early phase of the disease was troublesome and resulting in only fair discriminative performance of the model. However, we believed that as the model was derived entirely from the specific study domain intended to be used in outpatient practice, its performance should overcome previously developed criterion. Third, in terms of generalizability, the development cohort was based on highly endemic region of leptospirosis, so the application of OPD Lepto Score to non-endemic area might not be beneficial. Fourth, an external validation study is needed for evaluation of generalizability before the OPD Lepto Score being endorsed for real clinical use. Fifth, the confirmation of diagnosis other than leptospirosis in non-cases was not done. These some non-cases may be actual cases but remained undiagnosed because of various reasons including lesser severity which lowers diagnostic techniques sensitivity, higher antibiotic prescription (even non-significantly). These might increase the risk of false negatives.

12.Furthermore, some “non-cases” may be actual cases but remained undetected because of various reasons: for instance, non-cases seem less severe which lowers diagnostic techniques sensitivity; antibiotic prescription was higher in the non-cases even non-significantly, increasing the risk of false negatives; only half of patients had a convalescent sample for MAT, also increasing false negative cases…This undermines the robustness of the classification of the true negatives, and therefore the whole performance evaluation.

**Response:** We agreed with the Editor. We acknowledged this limitation in Discussion section, line 452-456, line 460-464.

“First, the study protocol planned to schedule all enrolled patients for 2^nd^ visit for blood samples for MAT to observe and interpret four-fold rising of convalescent titers, but only 48.9% of patients came for the blood tests. Thus, the number of confirmed cases could be underestimated in some degree.”

“Fifth, the confirmation of diagnosis other than leptospirosis in non-cases was not done. These some non-cases may be actual cases but remained undiagnosed because of various reasons including lesser severity which lowers diagnostic techniques sensitivity, higher antibiotic prescription (even non-significantly). These might increase the risk of false negatives.”

13.The conclusion is overstating the utility of the score (lines 390 and onwards). The mains limitations are not mentioned nor the need for studies to strengthen this first and initial evaluation. The term “validation” used by the authors for this score is an overstatement as well, as this study only provides a first and observational evidence. The design of the study indeed prevents from attributing any clear association between these indicators and the presence of *Leptospira*.

**Response:** We modified the final conclusion in the manuscript as follow “In conclusion, this study developed and internally validated the OPD Lepto Score, a practical clinical risk score for the diagnosis of leptospirosis in suspected patients with acute undifferentiated fever who presented to the outpatient clinics in high endemic areas. With 5 predictors, the score was more practical for outpatient setting than the conventional WHO score with 10 predictors.”, line 465-469.

For clarification, we used the term “development and internal validation”, which was not the same as “external validation”. If the term “external validation” was used, this would be in fact overstatement. With the term internally validate, we referred to the use of bootstrap resampling method to evaluate the presence of optimism and overfitting of the model. We also, already, acknowledged the importance of having future external validation study to assess for generalization of our score in other approximate setting in the last few sentences of the discussion section.

The TRIPOD guideline for transparent reporting of a multivariable prediction model for individual prognosis or diagnosis defined the importance of “internal validation” procedure as follow: The predictive performance of a model on the same data that was used to generate the results is referred to as the apparent performance of the model. Many prediction models are overfitted and their apparent performance optimistic, typically owing to the use of predictor selection strategies in small data sets. A better initial assessment of the performance of a prediction model is gained by assessing its performance using resampling techniques, such as cross-validation or bootstrapping, all referred to as internal validation. We recommend that all model development studies include some form of internal validation, particularly if no additional external validation is performed. For reference of TRIPOD, please follow this link: <https://annals.org/aim/fullarticle/2088542/transparent-reporting-multivariable-prediction-model-individual-prognosis-diagnosis-tripod-explanation>. We chose the best method for internal validation as suggested by the TRIPOD statement, an international widely-accepted reporting guidelines for clinical prediction rules development and validation.
